# Cranberry Proanthocyanidins-PANI Nanocomposite for the Detection of Bacteria Associated with Urinary Tract Infections

**DOI:** 10.3390/bios11060199

**Published:** 2021-06-19

**Authors:** Hilary Urena-Saborio, Anu Prathap M. Udayan, Emilia Alfaro-Viquez, Sergio Madrigal-Carballo, Jess D. Reed, Sundaram Gunasekaran

**Affiliations:** 1 Biosensors and Nanotechnology Laboratory, Department of Biological Systems Engineering, University of Wisconsin-Madison, 460 Henry Mall, Madison, WI 53706, USA; hilarydic93@hotmail.com (H.U.-S.); anuprathap@pec.edu.in (A.P.M.U.); 2 Department of Metallurgical and Materials Engineering, Punjab Engineering College (Deemed to be University), Sector-12, Chandigarh 160012, India; 3 Reed Research Group, Department of Animal Sciences, University of Wisconsin-Madison, 1675 Observatory Dr, Madison, WI 53706, USA; emilia.alfaro.viquez@gmail.com (E.A.-V.); sergio.madrigal.carballo@gmail.com (S.M.-C.); jdreed@wisc.edu (J.D.R.)

**Keywords:** proanthocyanidins, nanocomposites, urinary tract infections, extra-intestinal pathogenic *Escherichia coli*, bacteria agglutination

## Abstract

Consumption of cranberries is associated with the putative effects of preventing urinary tract infections (UTIs). Cranberry proanthocyanidins (PAC) contain unusual double A-type linkages, which are associated with strong interactions with surface virulence factors found on UTI-causing bacteria such as extra-intestinal pathogenic *Escherichia coli* (ExPEC), depicting in bacterial agglutination processes. In this work, we demonstrated the efficacy of cranberry PAC (200 μg/mL) to agglutinate ExPEC (5.0 × 10^8^ CFU/mL) in vitro as a selective interaction for the design of functionalized biosensors for potential detection of UTIs. We fabricated functionalized screen-printed electrodes (SPEs) by modifying with PAC-polyaniline (PANI) nanocomposites and tested the effectiveness of the PAC-PANI/SPE biosensor for detecting the presence of ExPEC in aqueous suspensions. Results indicated that the PAC-PANI/SPE was highly sensitive (limit of quantification of 1 CFU/mL of ExPEC), and its response was linear over the concentration range of 1–70,000 CFU/mL, suggesting cranberry PAC-functionalized biosensors are an innovative alternative for the detection and diagnosis of ExPEC-associated UTIs. The biosensor was also highly selective, reproducible, and stable.

## 1. Introduction

Urinary tract infection (UTI) is a public health concern, estimated to cost billions of dollars in diagnosis and treatment every year [1,2]. Consumption of cranberry has been correlated with the prevention of UTI, due to the presence of key plant metabolites called polyphenols. Among the different polyphenols found in cranberries, proanthocyanidins (PAC) possess special relevance for their actions against UTI bacteria. PAC are oligomers composed of flavan-3-ol monomers polymerized by interflavan bonds that can be described as either “A-type” (showing an additional C2-O-C7 ether bond) or “B-type” (showing a single C4-C6 or C4-C8 bond). The A-type linkages present in cranberry PAC are known to inhibit the adhesion of P-fimbriated *Escherichia coli* (*E. coli*) to uroepithelial cells [3].

Biosensors are a popular option for the detection of disease-causing agents, and when integrated with microfluidics, they can improve the rapid detection of UTI pathogens in urine samples for improved point-of-care treatment [4]. Currently, most UTI diagnoses are based on established techniques such as non-culturing and culturing methods, enzyme- and DNA-linked immunosorbent assay, mass spectrometry, and polymerase chain reaction (PCR), etc. [2,5]. These methods are time-consuming, expensive, and require specialized instrumentation for detection. Thus, the current trend in biosensor development is to seek direct detection of target bacteria in clinical samples, which will be a significant advantage over nucleic acid amplification techniques, such as PCR [6]. In this vein, some small molecules, including carbohydrates, tannins, lectin, and vancomycin, have emerged as important recognition elements for use in nanobiosensors. Due to their enhanced stability to temperature and pH variations, these small molecules have attracted attention for mediating interactions between nanomaterials and bacterial cells. These small recognition elements have a strong affinity to bind a broad range of bacterial cells, which are suitable for the detection of unanticipated bacteria. Compared with the use of antibodies or aptamers, the use of these small molecules provides a much higher density of recognition elements on the nanomaterial surface, and hence a strong affinity for the capture of bacterial cells [7].

Several studies have shown that naturally occurring chemicals such as PAC can interact with extra-intestinal pathogenic *E. coli* (ExPEC), which is the major cause of most varieties of extra-intestinal infections, including UTI, bacteremia, septicemia, surgical wound infections, and neonatal meningitis [8,9,10,11]. The structure–activity properties of cranberry PAC for interacting with surface virulence factors found on P-fimbriated ExPEC [12,13] led us to explore the use of PAC-ExPEC interaction for potential biosensing applications in diagnosing UTI. Therefore, this study was aimed at designing a biosensor for the detection of ExPEC, based on a hybrid nanocomposite matrix comprising A-Type PAC isolated from cranberry and nanofibers of conductive polymer polyaniline (PANI). The PAC possesses bacterial agglutination and anti-invasion properties against uropathogenic bacteria such as ExPEC, and PANI nanofibers offer high surface area and fast electron transfer. Therefore, the combined advantages of the PAC-PANI nanocomposites will afford improved biosensing of ExPEC, affording a fast response, high sensitivity, and reduced cost compared to many of the currently used methods.

## 2. Materials and Methods

### 2.1. Materials

Aniline monomer (C_6_H_5_NH_2_), ammonium persulfate (APS), (NH_4_)_2_S_2_O_8_, chloroform (CHCl_3_), hydrochloric acid (HCl), carbonyldiimidazole (CDI), and dimethylformamide (DMF) were purchased from Thermo Fisher Scientific (Waltham, MA, USA) and used without further purification. Screen-printed electrodes (SPE) were purchased from Zensor (supplied by CH Instruments, Inc., Austin, TX, USA). The ExPEC Strain-5011 used in this study was isolated from a clinical fecal sample and characterized to be genotypically representative of ExPECs by expressing both P and type 1 fimbriae [14,15]. The experimental results showed that the best current response was obtained when SPE was modified by drop-casting 4 µL of a catalyst ink (polyaniline).PAC-PANI/SPE showed good reproducibility of reduction peak current for calibration plot when using 0.1 M KCl solution containing 5 mM [Fe(CN)_6_]^3−/4−^. Other important parameters were optimized, such as pH, which was optimized to be 7.2. The results revealed that peak width = 0.2 s, pulse period = 0.5 s, and increment = 10 mV were found optimum for getting sharper and well-defined differential pulse voltammetry (DPV) peaks.

### 2.2. Biosensor Design and Fabrication

**Figure 1:**
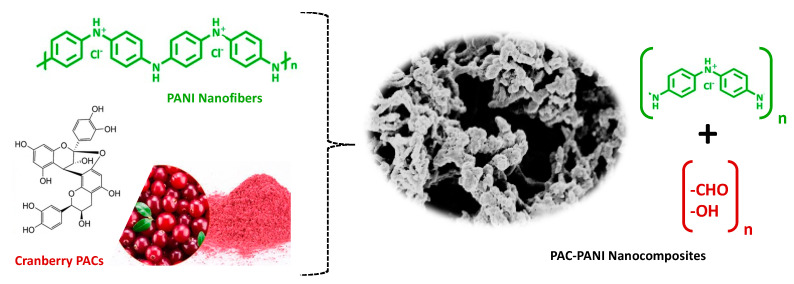
Cranberry PAC and PANI together yield a stable PAC-PANI nanocomposite, stabilized by hydrogen bonding and ion–dipole interaction.

We hypothesized that adsorption of PAC onto PANI nanofibers will increase sensitivity for detecting pathogenic bacteria associated with UTI, via the recognized interaction between PAC A-type interflavan bonds and surface virulence factors in ExPEC. Furthermore, polyphenolic moieties found in cranberry PAC will promote a favorable complexation with PANI’s amino groups, depicting PAC-PANI nanocomposites stabilized by highly dense hydrogen bonding and Van der Waals interactions (ion–dipole, dipole–dipole and π–π interactions), as illustrated in Figure 1. The interaction between PANI and polyphenols has been previously reported and successfully applied for the removal of phenolic compounds from aqueous solutions [16,17].

**Scheme 1:**
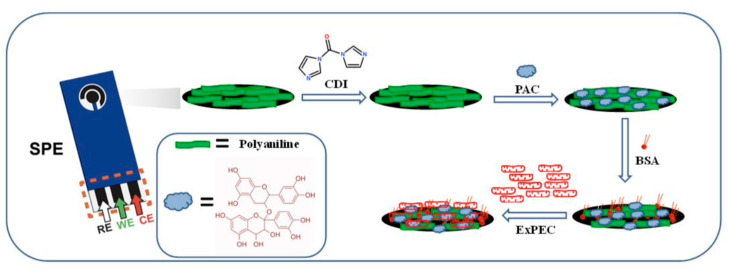
Scheme for electrochemical detection of UTI-causing extra-intestinal pathogenic *E. coli* (ExPEC) using a screen-printed electrode (SPE) functionalized with cranberry proanthocyanidins-polyaniline (PAC-PANI) nanocomposite.

The proposed mechanism of action of SPE electrodes functionalized with PAC-PANI nanocomposites is shown in Scheme 1. The biosensor was prepared by a two-step methodology. In the first step, PANI nanofibers were drop-cast on the SPE to obtain PANI/SPE, as previously described by Prathap et al. (2018) [18]. PANI nanofibers were prepared through a modified interfacial polymerization method at the interface of two immiscible solvents: chloroform and water. The SPE was coated by dropping 2 μL of nanofibers onto the working electrode surface of SPE and left to air dry for three hours. Then the electrode was activated with 0.5 M CDI for three hours at room temperature. In the second step, cranberry PAC was adsorbed onto the nanofibers to produce a PAC-PANI nanocomposite. The transduction concept is based on measurements of Faradaic impedance in the presence of the redox couple of [Fe(CN)_6_]^3−/4−^. Hexacyanoferrates are commonly used as redox probes for characterization. It is typically assumed that the electron transfer of hexacyanoferrates blocked by the formation of an ExPEC monolayer on the electrode surface, which is mediated by the presence of PAC on the working electrode.

The PAC was extracted from cranberries following the method of Feliciano et al. (2012) [19]. This involves nitrogen blending, solvent extraction, column chromatography, and freeze-drying. The freeze-dried PAC powder was characterized by HPLC and mass spectroscopy and quantified by the 4-(dimethylamino)cinnamaldehyde (DMAC) method. A 1 μL sample of PAC dissolved in DMF (20 mg/mL) was immobilized on the PANI/SPE and incubated overnight at 4 °C. To block nonspecific binding sites, the electrode was incubated with a solution of bovine serum albumin (BSA, 0.5%) for 15 min and washed three times with phosphate-buffered saline solution (PBS, pH = 7.2) to finally obtain PAC-PANI/SPE. 1,10-Carbonyldiimidazole (CDI) is known as the zero-length crosslinking reagent to couple hydroxyl functional groups with a primary amine. The covalent bonds between hydroxyl groups of PAC on the electrode surface and amine groups of PANI were formed with CDI chemistry. The mechanism is that CDI primarily reacts with the hydroxyl-containing PAC to form a reactive intermediate, imidazole carbamate, then subsequently reacts with amine-containing PANI, in which carbamate is created with the loss of imidazole. Therefore, CDI can be utilized for conjugation among organic compounds, peptides, and proteins, especially for their immobilization to a solid support [20].

### 2.3. Bacterial Agglutination Assay

The first step in biosensor design is to confirm the efficacy of PAC to bind to the target ExPEC. Therefore, the ability of cranberry PAC for binding to the surface virulence factors of ExPEC and agglutinate bacteria was tested by monitoring the increase in transmittance of light (450 nm) through a bacterial suspension with and without PAC, as described by Alfaro-Viquez et al. (2019) [21]. Briefly, 50 μL of ExPEC stock solution at 1.0 × 10^10^ colony forming units (CFU)/mL was added into a cuvette, achieving a final concentration of 5.0 × 10^8^ CFU/mL. Subsequently, 200 μg/mL of PAC was added to the bacterial inoculum and gently mixed. The absorbance of the sample was measured at 450 nm at 5 min intervals for 240 min using a spectrophotometer (DU 640, Beckman Coulter, IL, USA). The transmittance (%) and the area under the transmittance curve of the normalized data were calculated as a function of the ability of PAC to agglutinate ExPEC. 

### 2.4. Characterization of PAC-PANI/SPE and Bacterial Attachment

The solid-state behavior of a powdered PANI sample was evaluated by wide-angle X-ray diffraction (XRD). The XRD pattern was recorded with a scan speed of 2°/min on a PANalytical X’PERT PRO diffractometer (CuK-alpha radiation, λ = 0.1542 nm, 40 kV, 20 mA). UV–vis spectra were recorded on a Lambda 25 PerkinElmer spectrometer in the wavelength range of 200 to 800 nm with the PANI dispersed in deionized water. FTIR spectra were measured on a Bruker TENSOR-27 in the wavenumber range 400 to 4000 cm^−1^ at a 4 cm^−1^ resolution with KBr as compressed slices. The surface morphologies of the SPE functionalized by PANI and PAC-PANI were observed by scanning electron microscopy (SEM, Leo 1530-FE, Zeiss, Cambridge, UK). For bacterial adsorption studies, 100 μL of ExPEC at 10 CFU/mL was added onto the PAC-PANI/SPE followed by washing with PBS. The bacteria-treated PAC-PANI/SPE was also examined by SEM.

### 2.5. Electrochemical Measurements

A three-electrode electrochemical testing scheme was used with PAC-PANI/SPE with a 3 mm diameter carbon working electrode, carbon counter electrode, and silver/silver chloride reference electrode in a CHI-660D electrochemical workstation (CHI Instruments Inc., USA). The measurements were carried via DPV in 5 mm ferrocyanide/ferricyanide redox couple (K_3_[Fe(CN)_6_]/K_4_[Fe(CN)_6_]) in (1:1) solution with 0.1 M KCl (pH 7). The ExPEC were added in a dose-dependent manner from 1 to 70,000 CFU/mL onto the working electrode, and the resulting current was measured as a function of potential.

## 3. Results and Discussion

### 3.1. Material Characterization

**Figure 2:**
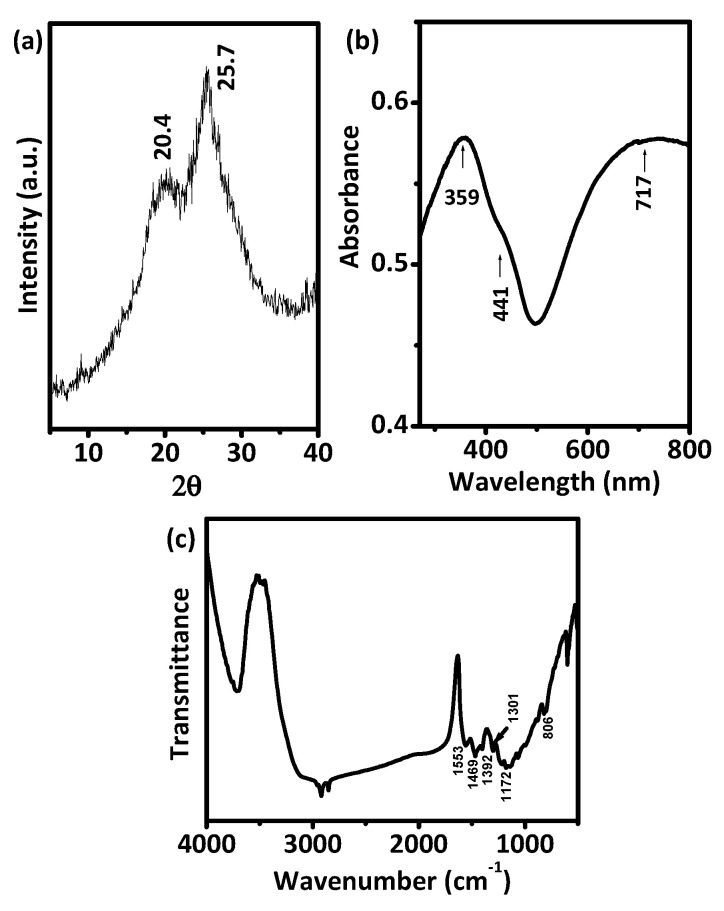
Characterization of PANI: (**a**) XRD pattern, (**b**) UV–visible spectrum, and (**c**) FTIR spectrum.

The XRD scan of PANI (Figure 2a) shows a peak centered at 2θ = 20.4°, which is credited to periodicity parallel to the polymer chain, and another peak at 2θ = 25.7°, which can be ascribed to periodicities perpendicular to the PANI chains. In the UV–vis absorption spectra of PANI, three characteristic absorption bands for doped PANI at wavelengths of 359, 441, and 717 nm are observed (Figure 2b). The absorption band at 359 nm is due to the π–π* transitions of a benzene ring and the bands at 441 nm and 717 nm are related to the doping level and polaron–bipolaron transition. The FTIR spectrum of PANI shows bands at 1553, 1469, 1392, 1172, and 806 cm^−1^ (Figure 2c). The bands observed at 1553 cm^−1^and 1469 cm^−1^correspond to C=C stretching of quinoid and benzenoid rings, and the bands at 1392 cm^−1^ and 806 cm^−1^ are assigned to N-H bending mode and out-of-plane deformation of C–H in the benzene ring, respectively. The peak around 1172 cm^−1^ is due to the degree of electron delocalization in PANI and stretching of N=Q=N in the quinoid (Q) ring.

### 3.2. ExPEC Agglutination

**Figure 3:**
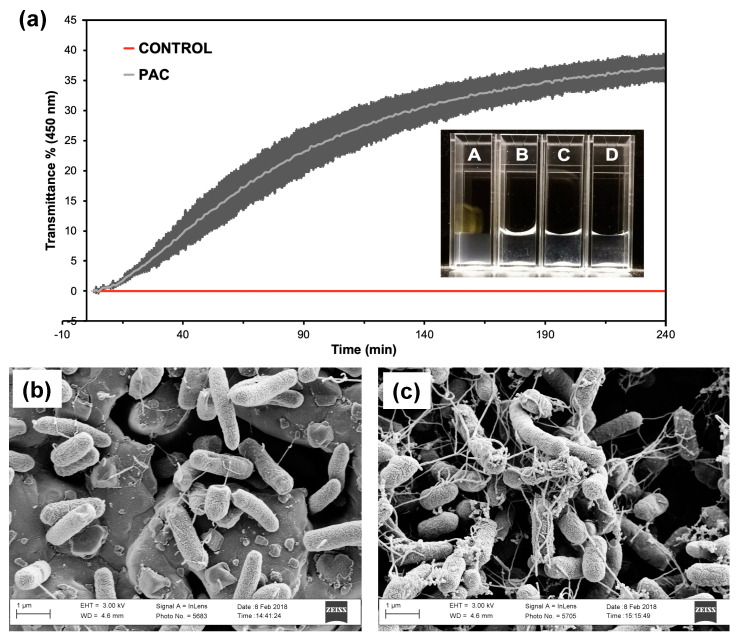
(**a**) UV–vis transmittance vs agglutination time for ExPEC (5.0 × 10 ^8^ CFU/mL) treated with and without cranberry PAC (200 μg/mL); dark grey area represents standard deviation values (n = 3). Inset: photograph of (**A**) control sample, in which ExPEC are dispersed and (**B**–**D**) PAC-treated (200 μg/mL) sample replicates, in which ExPEC are agglutinated and settling. SEM micrographs showing the surface morphology of (**b**) control, without PAC, and (**c**) PAC-treated (200 μg/mL) ExPEC, showing dense and entangled bacterial fimbrial tip structures.

As shown in Figure 3a, the light transmittance data from UV–vis results reveal that PAC (200 μg/mL) promotes agglutination of ExPEC when compared to the negative control (without PAC). The results are normalized to control, corresponding to ExPEC at 5 × 10^8^ CFU/mL, which, without the presence of PAC, does not show any precipitation and remains in solution, showing steady transmittance values (inset in Figure 3a(A)). As has been observed previously, PAC-ExPEC agglutination curves comprise two phases. The first phase, from 0 to ~25 min, corresponds to a lag phase, where the PAC begins to interact with surface virulence factors in ExPEC, forming a pseudo-colloidal suspension without noticeable agglutination; hence, the transmittance is rather poor. The second phase, from ~25 to 240 min, is associated with exponential agglutination and subsequent settling of ExPEC (inset in Figure 3a(B–D)), due to an increased PAC interaction with ExPEC, which leads to a significant increase in transmittance [21].

Several studies have shown that, after treatment with cranberry products, the PAC may act by blocking ExPEC strains from adhering to epithelial cells. The mechanism for this interaction has not been precisely identified, but research has revealed that the binding capacity of PAC to ExPEC is presumed to be driven by PAC’s attachment to fimbrial tips on the bacterial cell wall, which are involved in bacterial adhesion to uroepithelial cells [15,22,23,24,25]. SEM images of ExPEC, both untreated (Figure 3b) and treated (Figure 3c) with cranberry PAC (200 μg/mL), allow for identifying selective interactions between PAC-ExPEC. The presence of entangled fimbrial tips around most of the PAC-treated ExPEC body structures, which are not present in the control image corresponding to untreated ExPEC, show only the typical detangled fimbrial tips used for adhesion and colonization of uroepithelial cells. The unusual double A-type linkages present in the cranberry PAC may be important in the bacterial agglutination process [24]. Recent research has shown that higher agglutination of ExPEC and reduced bacterial invasion are correlated with a higher number of A-type bonds and a higher degree of polymerization of cranberry PAC [3,26].

**Figure 4:**
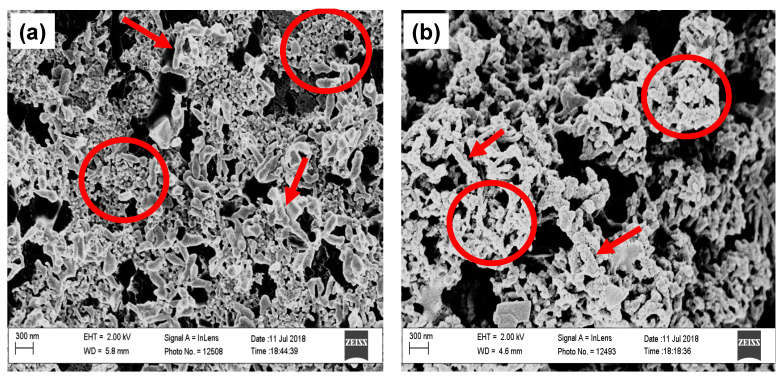
SEM micrographs showing the working electrode surface morphology of SPE modified by (**a**) PANI and (**b**) PANI-PAC. Arrows and circles are sections where morphological changes are visible upon the adsorption of PAC onto PANI, depicting the formation of PAC-PANI nanocomposites, as suggested by the coating-like appearance on the surface of nanofiber-like structures, and the reduction due to high-density coating in sections showing smaller nanoparticle-like structures, respectively.

The construction of our biosensor is based on PAC-PANI nanocomposites, stabilized by highly dense hydrogen bonding and Van der Waals interactions between polyphenolic moieties in cranberry PAC and amino groups in PANI. This affords a favorable complexation environment on the surface of an SPE when functionalized with PAC-PANI and leads to an optimal substrate for attachment and detection of bacteria associated with urinary tract infections. The efficacy of functionalization of the SPE sensor by PANI and PAC-PANI nanocomposites was characterized by SEM, as shown in Figure 4. SEM micrographs showed a change in the surface morphology of PANI nanofibers when interacting with PAC, suggesting a surface functionalization associated with the formation of PAC-PANI nanocomposites. The surface morphology of PANI (Figure 4a) shows the presence of small nanoparticle-like structures that are agglomerated among the surface (highlighted with circles) and other sections showing longer fiber-like structures (highlighted with arrows). Once the PANI has interacted with PAC, the formation of PAC-PANI nanocomposites can be associated with the presence of surface changes on the SEM image, suggesting coating of the PANI nanostructures upon addition of PAC. The circled sections of the PAC-PANI nanocomposites show a visible reduction in nanoparticle-like structures that evolved onto a highly dense coated surface, similar to the changes observed for the nanofiber-like structures highlighted by arrows (Figure 4b).

### 3.3. Sensor Performance

**Figure 5:**
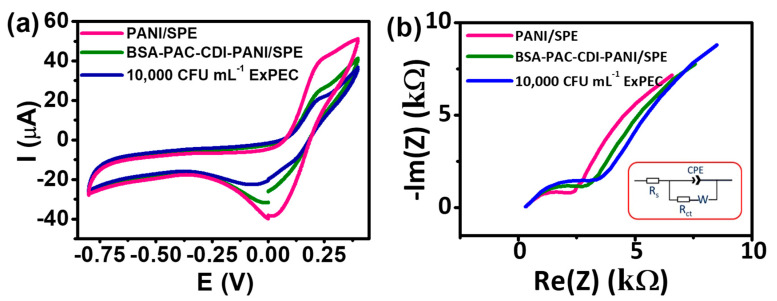
CV (**a**) and EIS spectra (**b**) of PANI/SPE, BSA-PAC-CDI-PANI/SPE, and 10,000 CFU mL^−1^ ExPEC in 0.1 M KCl solution containing 5 mM [Fe(CN)_6_]^3-/4-^. The inset of (**b**) shows an equivalent circuit model.

**Figure 6:**
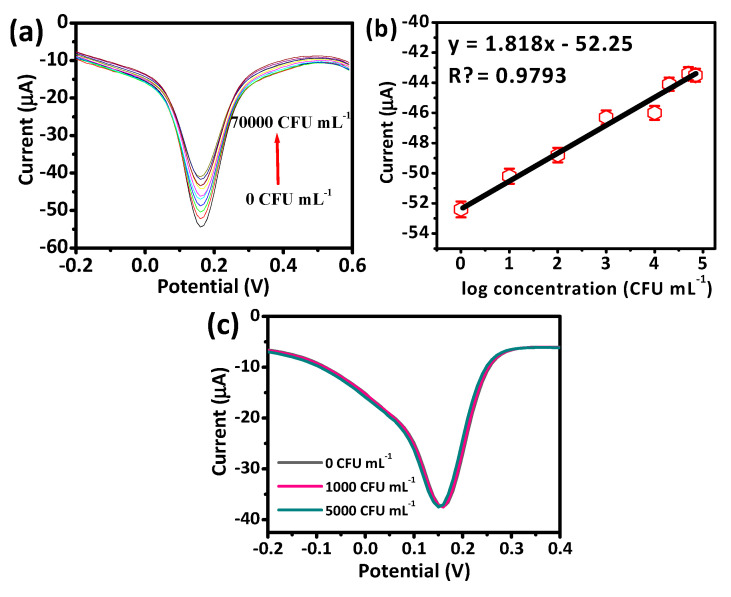
(**a**) DPV curves at varying concentrations of ExPEC from 0 to 70,000 CFU/mL at PAC-PANI/SPE in 0.1 M KCl solution containing 5 mM [Fe(CN)_6_]^3-/4-^. (**b**) Calibration curves of peak current versus the logarithmic value of ExPEC concentration. (**c**) DPV curves at various concentrations of ExPEC at PANI/SPE in 0.1 M KCl solution containing 5 mM [Fe(CN)_6_]^3-/4-^.

A biosensor for the detection of ExPEC was made using PAC-PANI/SPE. The electrodes at different fabrication steps were characterized by cyclic voltammetry (CV) and electrochemical impedance spectroscopy (EIS)using [Fe(CN)_6_]^3−/4−^ as the probe (Figure 5). A pair of reversible redox peaks for the redox of [Fe(CN)_6_]^3−/4−^was observed in the voltammogram in Figure 5 at PANI/SPE with a redox peak separation (∆*E*) of 231.4 mV, and the charge transfer resistance (R_ct_) is estimated to be 2371 Ω (Table 1). For the facile immobilization of BSA-PAC-CDI on the SPE surface, the values of ∆E and R_ct_ are 241 mV and 2893 Ω. The substantially increased ∆E compared with those for PANI/SPE indicates the successful modification of PANI. The effective capture of 10,000 CFU mL^−1^ shows a further increased ∆*E* (280 mV) and 3537 Ω. The sensitivity of the PAC-PANI/SPE for detecting bacteria associated with UTI was assessed by measuring the DPV current responses at different ExPEC concentrations (Figure 6a). The calibration plot of peak current response vs. ExPEC concentration (1 to 70,000 CFU/mL) shows an excellent log-linear relationship (Figure 6b). This result suggests that our biosensor was able to detect the presence of ExPEC at a concentration as low as 1 CFU, demonstrating ultralow sensitivity. Based on three consecutive DPV measurements, LOQ (lowest analyte concentration necessary to generate a characteristic signal at least 10-fold higher than the background noise) [27] was calculated as 1 CFU/mL. The PANI/SPE sensor was non-responsive to ExPEC (Figure 6c). The comparison of the outcome to the LOD of targeted bacteria for some biosensor methods reported in the literary works (Table 2) permits us to conclude that our system is more suitable for bacteria detection.

**Figure 7:**
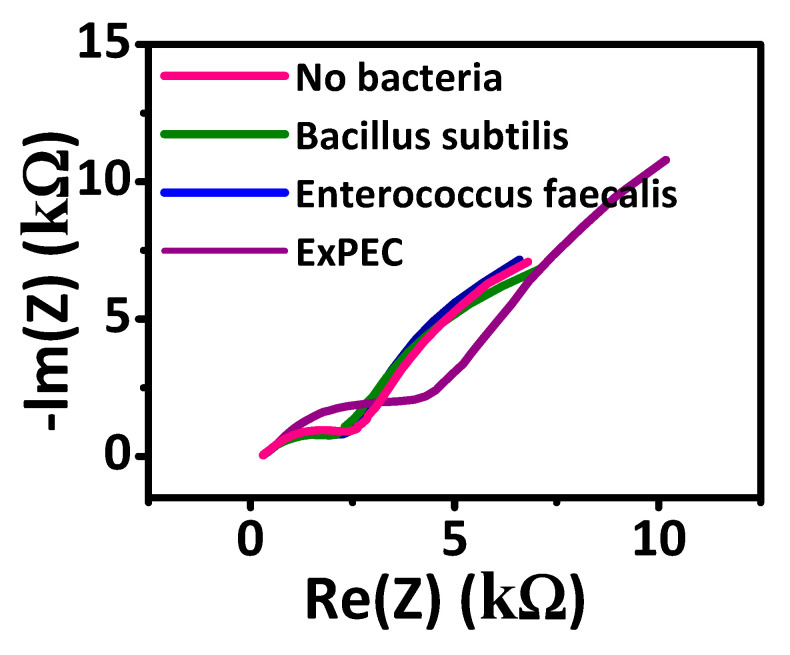
EIS study for *E. faecalis* (4.0 × 10^3^ CFU mL^−1^), *B. subtilis* (4.0 × 10^3^ CFU mL^−1^), and ExPEC (4.0 × 10^3^ CFU mL^−1^).

**Figure 8:**
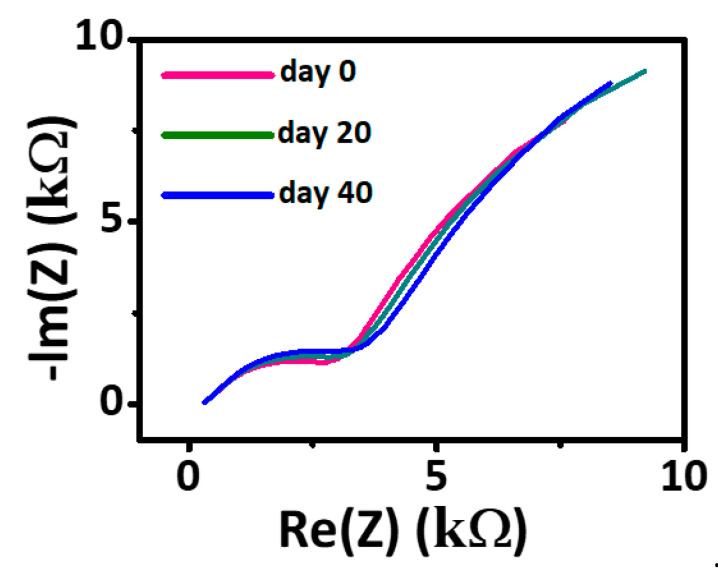
Stability of the PAC-PANI/SPE biosensor over 40days in refrigerated storage at 4 °C.

To assess the selectivity of our biosensor, the detection of *Enterococcus faecalis* (*E. faecalis*) (4.0 × 10^3^ CFU mL^−1^), *Bacillus subtilis* (*B. subtilis*) (4.0 × 10^3^ CFUmL^−1^), and ExPEC (4.0 × 10^3^ CFU mL^−1^) were evaluated. As shown in Figure 7, the impedance signal for ExPEC is significantly higher than those for *E. faecalis and B. subtilis*, suggesting the interference of other bacteria is negligible. The reproducibility of the sensor was checked by fabricating three different SPEs for the detection of 3.0 × 10^3^ CFU/mL of ExPEC. The RSD of the measurements for the three electrodes was 3.9%. The excellent reproducibility could be due to the PAC and also the sensor design and fabrication approaches. A similar test was additionally done, but utilizing a batch of three SPEs and kept in the refrigerator at 4 °C. The electrode was evaluated to 3.0 × 10^3^ CFU/mL of ExPECfor40 days. The electrochemical feedback was found to decrease by only 9.8% after 40 days, showing that the sensor surface is stable (Figure 8).

To evaluate the practical feasibility of our electrochemical assay, ExPEC was detected in commercial city water and milk samples. The samples were diluted by PBS and were spiked with ExPEC by the standard addition method with final concentrations of 2.0 × 10^3^ and 2.0 × 10^4^ CFU/mL. As can be seen from Table 3, the recoveries were 94 and 96% in water and 86 and 82% in milk. The fat, protein, and other substances present in milk samples are attributed to the lower recovery in milk than in water.

**Figure 9:**
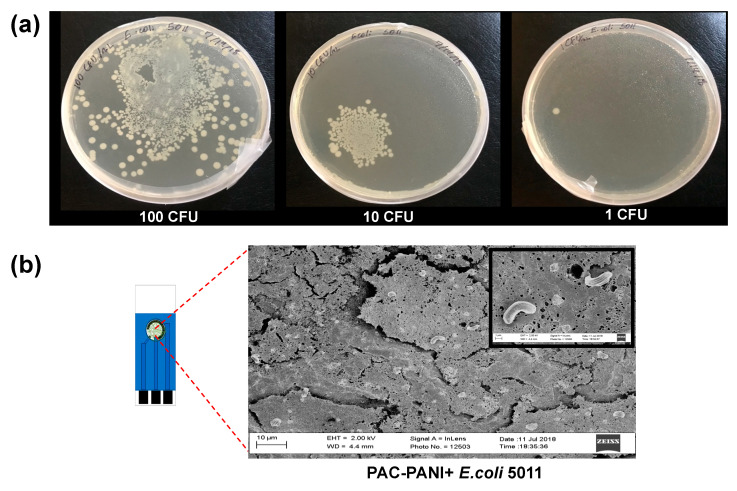
(**a**) Plate culturing of the ExPEC at 100, 10, and 1 CFU/mL loads. (**b**) SEM micrograph showing the adhesion ExPEC on the functionalized surface of the working electrode of PAC-PANI/SPE after successive washings with PBS.

Culture plates in Figure 9a show the corresponding bacterial concentrations used for the frequency response study and confirm the ultrasensitive response of the biosensor to low concentrations of UTI-associated bacteria. The SEM image of the PAC-PANI/SPE biosensor after detection experiments showed the presence of adsorbed ExPEC structures embedded in the PAC-PANI nanocomposite surface (Figure 9b). These bacteria remained attached to the biosensor after successive washings with PBS, suggesting a high affinity of the functionalized biosensor for adsorption of ExPEC onto PAC-PANI nanocomposites and depicting ultrasensitive LOQ values for the detection of UTI bacteria. This may result from the intrinsic structure of the PAC-PANI nanocomposites biosensor, which was able to entrap a high number of PAC molecules and conserve their active structure. On the other hand, the PAC affinity to ExPEC promotes efficient adsorption of ExPEC and high sensitivity for detection [35,36]. Thus, high DPV activity was achieved by the functionalized biosensor due to the positive interaction of the SPE and the immobilization of the PAC-PANI nanocomposite matrix with ExPEC. To our understanding, this study constitutes the first approach to evaluate the potential of cranberry PAC as a recognition element in biosensors for the detection of ExPEC associated with UTIs. The ultra-high sensitivity of PAC for ExPEC supports the possibility that the future UTI diagnostics based on PAC-based biosensors, in addition to being faster, will also be more informative than the current approaches, allowing the detection in patients with asymptomatic bacteriuria compared to true UTI, achieved by detecting the presence of ultralow amounts of bacteria in urine samples.

## 4. Conclusions

We have demonstrated that cranberry PAC has a specific affinity for ExPEC that enables their agglutination and thus can be used for the detection of bacterial pathogens associated with UTI. Agglutination experiments and SEM imaging suggest that PAC may bind to ExPEC through strong interactions with fimbrial tips on the bacterial cell wall. We fabricated an electrochemical biosensor by functionalizing the working electrode of an SPE with PAC-PANI nanocomposites, stabilized by highly dense hydrogen bonding and Van der Waals interactions between polyphenolic moieties in cranberry PAC and amino groups in PANI, affording a favorable complexation environment. The functionalized PAC-PANI electrode was able to successfully bind ExPEC and produced DPV current signals. This response was not observed with SPE functionalized just with PANI (without PAC) and ExPEC, indicating a bioconjugation mechanism unique to PAC-ExPEC interactions. The sensor response was linear over the ExPEC range of 1 to 70,000 CFU/mL, and it exhibited ultralow sensitivity with a limit quantification (LOQ) of 1 CFU/mL. The sensor showed excellent selectivity and also outstanding stability. The successful detection of ExPEC in real matrices such as water and milk demonstrates the potential practical use of this biosensor.

## 5. Patents

J.D. Reed: C. G. Krueger, E. Alfaro-Viquez, S. Madrigal-Carballo, H. Urena-Saborio, S. Gunasekaran.“Tannin composite fibers” US Patent Application # P190308US01 (June 2019).

## Figures and Tables

**Table 1:** Components of Randles equivalent circuit at various stages of surface modifications of the SPE.

Sample	R_sol_ (Ω)	R_ct_ (Ω)	Z_0_ (µF cm^−2^)	W (Ω cm^2^)
PANI/SPE	26.3	2371	18.3	3.2 × 10^−^^4^
BSA-PAC-CDI-PANI/SPE	19.3	2893	14.3	2.8 × 10^−^^4^
10,000 CFU mL^−1^ ExPEC	9.3	3537	10.2	1.9 × 10^−^^4^

**Table 2:** Comparison between the proposed method and bacterial biosensors reported.

Reference	Immobilization Method	Limit of Detection	Linear Range
[28]	immobilization of antibody via cross-linking heterobifunctional	10^4^ CFU/mL	10^4^–10^7^ CFU/mL
[29]	anchoring of antibodies by epoxysilane	6 × 10^3^ cells/mL	6 × 10^4^–6 × 10^7^ cells/mL
[30]	anchoring of yeast cells	10^2^ CFU/mL	10^2^–10^8^ CFU/mL
[31]	anchoring of antibodies by epoxysilane	6 × 10^5^ cells/mL	-
[32]	immobilizing anti-*E. coli* antibodies	6 × 10^6^ CFU/mL	4.36 × 10^5^–4.36 × 10^8^ CFU/mL
[33]	anchoring of antibodies by epoxysilane	4.215 × 10^3^ CFU/mL	4.215 × 10^3^–4.215 × 10^6^ CFU/mL
[34]	magnetic nanoparticle antibody conjugates	7 × 10^4^ CFU/mL	-
This work	PAC-polyaniline (PANI) nanocomposites	1 CFU/mL	1–70,000 CFU/mL

**Table 3:** Detection results and recoveries in water and milk samples.

Sample	Spiked (CFU mL^−1^)	Found (CFU mL^−1^)	Recovery (%)
Water 1	2.0 × 10^3^	1.88 × 10^3^	94.0
Water 2	2.0 × 10^4^	1.92 × 10^3^	96.0
Milk1	2.0 × 10^3^	1.72 × 10^3^	86.0
Milk2	2.0 × 10^4^	1.64 × 10^3^	82.0

## Data Availability

Not applicable.
